# The relationship between healthy lifestyles and cognitive function in Chinese older adults: the mediating effect of depressive symptoms

**DOI:** 10.1186/s12877-024-04922-5

**Published:** 2024-03-28

**Authors:** Guowei Xian, Yulin Chai, Yunna Gong, Wenfeng He, Chunxiao Ma, Xiaolin Zhang, Jing Zhang, Yong Ma

**Affiliations:** School of Management, Shandong Second Medical University, 261053 Weifang, Shandong China

**Keywords:** Healthy lifestyles, Cognitive function, Depressive symptoms, Mediating effect, Older people

## Abstract

**Background:**

Previous studies have proven the positive relationship between healthy lifestyles and cognitive function in older adults. However, the specific impacts and mechanisms require further investigation. Therefore, this study aimed to investigate whether healthy lifestyles and cognitive function were associated with Chinese older adults and whether depressive symptoms mediated their association.

**Methods:**

8272 valid samples were included using the latest data from the Chinese Longitudinal Healthy Longevity Survey (CLHLS). Pearson’s test was applied to investigate the relationship between the key variables. Regression models were employed to examine the mediating effects of healthy lifestyles, using Sobel’s test and the bootstrap method to confirm path effects.

**Results:**

There was a significant correlation between healthy lifestyles, depressive symptoms, and cognitive function (*p* < 0.01). Healthy lifestyles directly impact cognitive function (β = 0.162, *p* < 0.01). Healthy lifestyles had a significant effect on depressive symptoms (β=-0.301, *p* < 0.01), while depressive symptoms have a significant impact on cognitive function (β=-0.108, *p* < 0.01). Depressive symptoms partially mediated the effect of healthy lifestyles on cognitive function (β = 0.032, *p* < 0.01). The Sobel and bootstrap tests confirmed the robustness of the regression analysis results.

**Conclusion:**

Depressive symptoms mediate the relationship between healthy lifestyles and cognitive function. Our findings suggest that prevention strategies for cognitive impairment in older adults should focus on healthy lifestyles and mental health.

## Introduction

Population aging poses a significant challenge to public health worldwide. China, in particular, is currently undergoing a rapid and profound aging process [1]. According to China’s population census data, the total number of individuals aged 60 and older in China has reached 264 million, accounting for about 18.7% of the total population. Concurrently, cognitive decline associated with aging has emerged as a growing public health concern [2]. Some studies have shown that there are nearly 15 million dementia patients in China, approximately 25% of the worldwide total, and this number is constantly increasing [3]. Impaired cognitive function was associated with a range of adverse health outcomes, including mild cognitive impairment, Alzheimer’s disease, depressive symptoms, and physical impairment [4]. Furthermore, the decline in cognitive function carries serious social and economic burdens [5]. It is estimated that cognitive impairment results in a greater disease burden compared to cancer and cardiovascular disease, equating to approximately $19,144.36 per patient [6]. Therefore, addressing age-related cognitive decline has become a priority for public health [7].

The World Health Organization (WHO) has defined healthy lifestyles as taking actions that individuals can take within their capacity to promote physical, mental, and social health [8]. According to WHO recommendations, smoking, alcohol consumption, diet, and physical activity are identified as the four primary lifestyle behaviors influencing health outcomes [9]. Increasing evidence suggests that healthy lifestyles are important protective and easily modifiable factors for cognitive function [10]. Therefore, numerous researchers have explored the relationship between healthy lifestyles and cognitive function in different populations. For instance, a systematic review has demonstrated that individuals who abstain from smoking exhibit superior performance in daily activities and specific cognitive tasks compared to smokers [11]. Research has established that long-term excessive alcohol consumption may impair an individual’s executive function, memory, and metacognitive abilities [12]. Findings from a study conducted in China have revealed that adopting healthier lifestyles, which encompass abstaining from smoking and alcohol, engaging in moderate exercise, and maintaining a healthy diet and weight, is associated with enhanced cognitive function after accounting for genetic factors [13]. Similarly, a longitudinal study involving Korean elderly individuals found that maintaining an overall healthy lifestyle, measured by a composite healthy lifestyle score derived from factors such as smoking, alcohol consumption, weight, and exercise, was effective in preventing or mitigating cognitive decline [14]. Therefore, healthy lifestyles are an essential factor in preventing and mitigating cognitive impairment in older adults.

Depressive symptoms are a common mental disorder with significant impacts on an individual’s health and daily life [15]. Previous studies have shown that positive, healthy lifestyles are associated with a reduced risk of depressive symptoms [16, 17]. For example, a cross-sectional study found that higher smoking frequency and volume were associated with an increased risk of depressive symptoms in older adults [18]. A multinational study encompassing more than 50 countries identified a connection between alcohol consumption and episodes of depressive symptoms, though these associations exhibited heterogeneity across different nations [19]. Physical exercise could produce antidepressant effects through multiple biological and psychosocial pathways. Downstream cellular processes stimulated by neurotrophins during physical activity could result in structural changes in the brain, such as improved vascular circulation, which in turn improves areas associated with depressive symptoms, such as the hippocampus [20]. Physical activity probably increases personal social interactions and social support [21], contributing to a reduced risk of depressive symptoms [22]. Furthermore, several studies have emphasized the importance of dietary patterns and dietary diversity for the health of older adults, including mobility, physical health, and depressive symptoms [23, 24]. Therefore, positive and healthy lifestyles play a pivotal role in ameliorating depressive symptoms.

The relationship between depressive symptoms and cognitive function has been extensively studied in recent years [25]. Cognitive impairment is a central feature of depressive symptoms and is mainly characterized by a significant trend of decline in executive functioning, memory, and attention in depressed patients [26]. Cognitive function naturally diminishes in older adults, influenced by various factors, including structural changes in the brain (such as fewer neurons and changes in synaptic connectivity), cerebrovascular disease, medication side effects, reduced physical activity, and psychological and social stress [27]. Notably, depressive symptoms further exacerbate the adverse impact of these contributing factors [28]. Furthermore, some studies have proposed that depressive symptoms might serve as early indicators or precursors of cognitive decline, potentially contributing to subsequent cognitive deterioration in older adults [29]. For instance, a longitudinal study has established an association between depressive symptoms and diminished cognitive function in older adults [30]. Individuals experiencing depressive symptoms often suffer from low self-esteem, decreased motivation, and disruptions in sleep patterns, all of which can impose a cognitive burden [31]. Moreover, poorer sleep quality may impede the brain’s ability to efficiently remove protein waste, potentially leading to cognitive decline [32]. Therefore, depressive symptoms are an important predictor of cognitive function in older adults.

An integrated healthy lifestyle considers the interactions and interconnectedness between different lifestyles among various aspects of healthy living rather than concentrating solely on a single facet of a healthy lifestyle [33]. Therefore, it is important to analyze the potential effects of integrating healthy lifestyles on the cognitive performance of older adults [34]. Moreover, most of the existing evidence comes from studies conducted in developed countries, making it challenging to generalize the findings to other populations due to variations in genetic factors, economic disparities, and sociocultural distinctions [35]. Given that China is the world’s largest developing country and has the largest elderly population, it is imperative to conduct extensive investigations into the healthy lifestyles and cognition of Chinese older adults [36]. Several scholars have investigated the relationship between healthy lifestyles and cognitive function in older adults [37, 38]. However, few have investigated the mediating role of depressive symptoms between healthy lifestyles and cognitive function among older adults in the Chinese context. Therefore, this study aimed to investigate the relationship between healthy lifestyles, depressive symptoms, and cognitive functioning in older adults. Based on the theories and literature above, we proposed the following hypotheses:

H1: Cognitive function is influenced by healthy lifestyles.

H2: Depressive symptoms are influenced by healthy lifestyles.

H3: Cognitive function is influenced by depressive symptoms.

## Methods

### Study population and data source

**Fig. 1:**
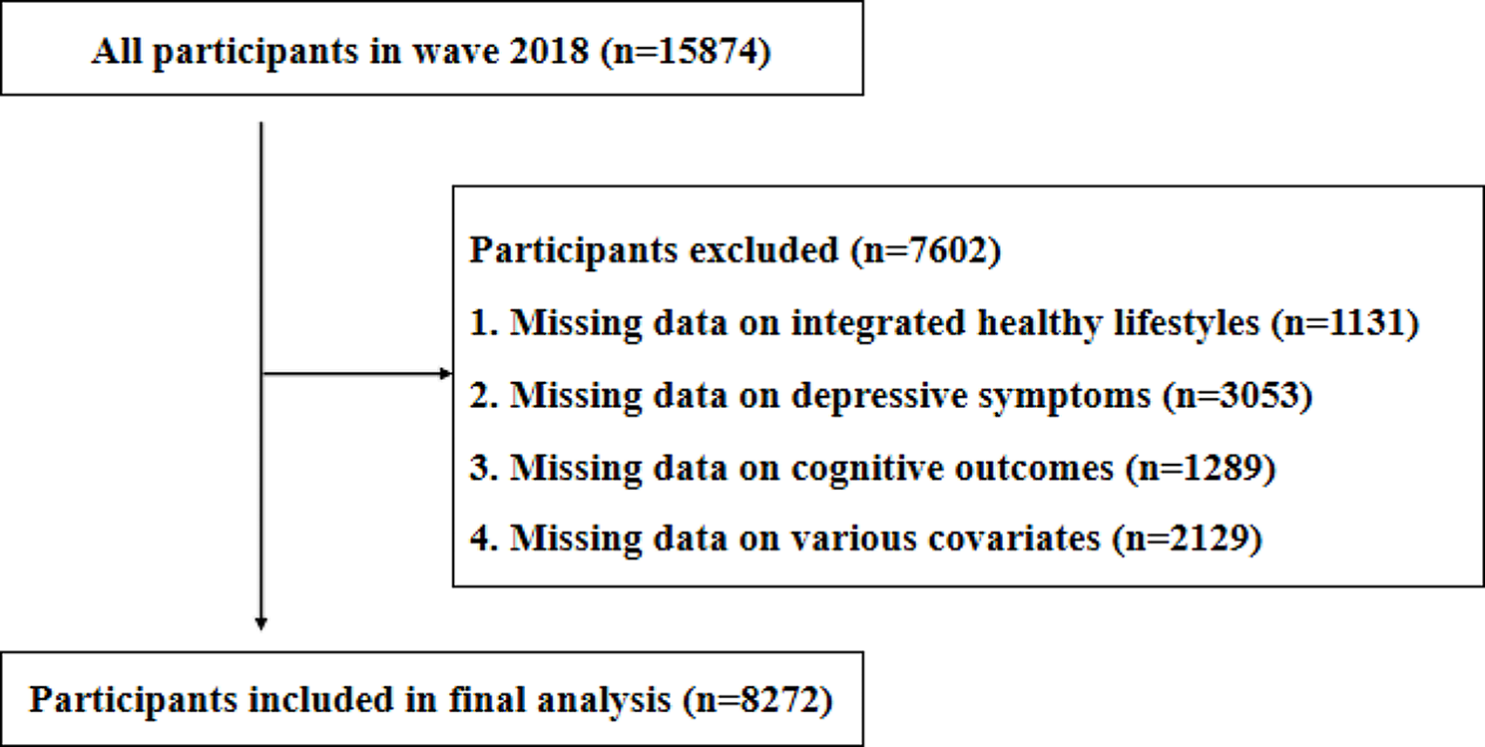
Absence of relevant important variables

The data for this study were derived from the 2018 wave of the Chinese Longitudinal Healthy Longevity Survey (CLHLS). The CLHLS is a nationally representative comprehensive survey of the elderly in China, covering the domains of physical and mental health, cognitive function, social participation, health behaviors, socioeconomic status, family structure, intergenerational relationships, and caregiving needs. The CLHLS was initiated in 1998 and carried out every two to three years by Peking University’s Center for Healthy Aging and Family Studies and the China National Research Center on Aging. To ensure good scientific and national representativeness, CLHLS employed a multi-stage disproportionate and targeted random sampling method covering 23 provinces, municipalities, and autonomous regions in China. We excluded samples with ages less than 60 and missing variables. A total of 8272 observations were finally included in the final analysis. The absence of relevant important variables is shown in Fig. 1.

Every participant provided written informed consent. CLHLS was approved by the Research Ethics Boards of Peking University and Duke University (IRB00001052-13074) and conducted in accordance with approved guidelines. This study utilized publicly available and de-identified data from CLHLS 2018, thus exempting it from the need for additional ethical approval.

### Measures

Referring to previous studies [39], a healthy lifestyle was measured by smoking, drinking, exercise status, and nutritional intake. Smoking, drinking, and not exercising were scored as zero points. Otherwise, they were scored as one point. Nutritional intake was assessed using the Dietary Diversity Score (DDS), which considered consumption of fruits, vegetables, meat, eggs, fish, beans, nuts, milk, and tea. The DDS scale has acceptable reliability (Cronbach’s alpha = 0.631) and validity (KMO = 0.747, *p* < 0.001). Individuals who responded “often or almost every day” were scored as one, while those who reported “occasionally” or “rarely or never” were scored as zero. The DDS was calculated by summing the scores of all the items, resulting in a range from 0 to 9, where higher scores signified greater dietary diversity. A score of 1 was assigned for a favorable DDS (7–9 DDS); otherwise, a score of 0 was recorded [40]. Consequently, we summed scores for smoking, drinking, physical activity, and DDS to calculate the Healthy Lifestyle Score. The Healthy Lifestyle Score ranged from 0 to 4, with higher scores indicating healthier lifestyles.

Depressive symptoms were measured using the Center for Epidemiologic Studies Depression Scale (CESD-10) [41]. The CESD-10 exhibited good reliability and validity, which was validated and applied to Chinese older adults. In this study, the reliability (Cronbach’s alpha = 0.735) and validity (KMO = 0.839, *p* < 0.001) of the CES-D10 scale were good. During the process of the CES-D10 investigation, participants were requested to assess their personal status in the past week. Each question had four options: 0 = never, 1 = seldom or sometimes, 2 = sometimes, and 3 = always. The CESD-10 score was derived by summing the responses to the 10 items, ranging from 0 to 30. Higher CESD-10 scores were associated with more severe depressive symptoms [42].

Cognitive function in the CLHLS study was evaluated using the Chinese version of the Mini-Mental State Examination (MMSE), which has been validated for validity and reliability [43]. The MMSE questionnaire comprised 24 items that evaluated orientation, one-minute food counting, attention, calculation, drawing, recall, and language skills. Food counting in one minute was scored from 0 to 7 points, with 1 point assigned for each correctly counted food item, up to a maximum of 7 points. Other questions were assigned a value of 1 for a correct answer and 0 for an incorrect answer. MMSE scores ranged from 0 to 30, with higher scores denoting higher cognitive function. Participants with MMSE scores ≥ 24 and < 24 are respectively defined as having “normal cognitive function” and “cognitive impairment [44]. In this study, the MMSE scale had good reliability (Cronbach’s alpha = 0.705) and validity (KMO = 0.818, p < 0.001) [45].

Considering the effect of potential confounders, several covariates were included in this study, including age, gender, ethnicity, household registration, marriage status, years of education, socioeconomic status, living arrangements, and self-rated health. Gender, ethnicity, hukou, marital status, and living arrangements were dichotomous variables. Living arrangements are categorized as living alone and living with household member(s). Residence with household member(s) was set to 1, otherwise it was 0. Socioeconomic status and self-assessed health were scored using a five-point Likert scale, with responses ranging from 1 (very poor) to 5 (very good). Years of education were assessed based on the question, “How many years of schooling have you had?”

### Statistical analysis

We conducted all statistical analyses using STATA version 17.0. Firstly, this study performed a statistical description of sociodemographic characteristics and key variables. Categorical variables were described by frequencies (percentages) and continuous variables by means (standard deviation, SD). Differences in characteristics between subgroups of different cognitive functions were compared using t-tests or chi-square tests [46]. Secondly, Pearson’s correlation analysis was employed to measure bivariate correlations between healthy lifestyles, depressive symptoms, and cognitive function. Next, Judd and Kenny’s recommendations and Baron and Kenny’s causal step approach were employed to investigate the relationship between healthy lifestyles, depression, and cognitive function in older adults [47]. Therefore, the three regression processes would estimate the following effects: (1) the effect of healthy lifestyles on cognitive function; (2) the effect of healthy lifestyles on depressive symptoms; (3) the effect of depressive symptoms on cognitive function when healthy lifestyles were controlled; and (4) the effect of healthy lifestyles on cognitive function when depressive symptoms were controlled. Covariates were included in all regression analyses to improve the accuracy of model estimation. Subsequently, we used the Sobel test to rigorously examine whether the indirect effect was significantly non-zero. The indirect effect was considered significant if the Z-value obtained from the Sobel test exceeded 1.96. To determine effect sizes and obtain 95% confidence intervals, we assessed direct, indirect, and total effects using the bootstrap method with 5000 iterations [47]. A significant effect was shown when the 95% confidence interval did not include zero. All statistical tests were two-sided, and *p <* 0.05 was considered statistically significant.

## Results

Table 1 presents the descriptive statistics of the participants. A total of 8272 participants were recruited in the study, with an average age of 81.73 years, comprising 3954 males and 4318 females. The majority of participants were Han Chinese (94.79%), divorced, widowed, or unmarried (51.06%), registered rural households (67.70%), and lived with others (83.64%). Participants whose socio-economic status was very poor, poor, fair, good, and very good accounted for 0.85%, 11.74%, 38.32%, 35.88%, and 3.02%, respectively. The percentages of participants who rated their health as very poor, poor, fair, good, and very good were 1.05%, 8.09%, 69.73%, 18.11%, and 13.21%, respectively. The means for education, healthy lifestyles, depressive symptoms, and cognitive function were 4.07 years, 2.28 points, 7.15 points, and 26.79 points, respectively. There were significant differences between cognitive impairment and normal cognitive functioning in terms of age, gender, ethnic, marriage, years of education, household registration, self-rated health, socioeconomic status, healthy lifestyles, depressive symptoms, and cognitive functioning.

**Table 1:** Sociodemographic characteristics and critical variables of participants

Variable	Overall (*N* = 8272)	Cognitive impairment		*P* value
		No (*N* = 6967)	Yes (*N* = 1305)	
**Age(years)**	81.73 (10.97)	79.98 (10.30)	91.06 (9.65)	0.00
**Gender**				0.00
Female	4318 (52.20)	3422 (49.12)	896 (68.66)	
Male	3954 (47.80)	3545 (50.88)	409 (31.34)	
**Ethnic**				0.03
Non-Han	431 (5.21)	379 (5.44)	52(3.98)	
Han	7841 (94.79)	6588 (94.56)	1253(96.02)	
**Marriage**				0.00
divorced, widowed, or unmarried	4224 (51.06)	3211 (46.09)	1013 (77.62)	
Married	4048(48.94)	3756 (53.91)	292 (22.38)	
**Years of education**	4.07(4.46)	4.55 (4.51)	1.48 (3.05)	0.00
**Household registration**				0.00
Rural	5600(67.70)	4592 (65.91)	1008 (77.24)	
Urban	2672(32.30)	2375 (34.09)	297 (22.76)	
**Self-rated health**				0.00
Very poor	70(0.85)	46 (0.66)	24 (1.84)	
Poor	971(11.74)	768 (11.02)	203 (15.56)	
Fair	3170(38.32)	2624 (37.66)	546 (41.84)	
Good	2968(35.88)	2551 (36.62)	417 (31.95)	
Very good	1093(13.21)	978 (14.04)	115 (8.81)	
**Socioeconomic status**				0.00
Very poor	87(1.05)	68 (0.98)	19 (1.46)	
Poor	669(8.09)	523 (7.51)	146 (11.19)	
Fair	5768(69.73)	4827 (69.28)	941 (72.11)	
Good	1498(18.11)	1324 (19.00)	174 (13.33)	
Very good	250(3.02)	225 (3.23)	25 (1.92)	
**Living arrangement**				0.31
alone	1353(16.36)	1127 (16.18)	226 (17.32)	
with household member(s)	6919(83.64)	5840 (83.82)	5840 (82.68)	
**Healthy Lifestyles**	2.28(0.89)	2.30 (0.92)	2.14 (0.70)	0.00
**Depressive symptoms**	7.15(4.39)	6.85 (4.24)	8.75 (4.83)	0.00
**Cognitive function**	26.79(4.17)	28.27 (1.80)	18.90 (4.34)	0.00

As shown in Table 2, the results of Pearson’s correlation analysis suggested that there were mutually significant correlations between healthy lifestyles, depressive symptoms, and cognitive function (*p <* 0.01). Specifically, healthy lifestyles were negatively associated with depressive symptoms (*r*=-0.102, *p <* 0.01) and positively associated with cognitive function (*r* = 0.084, *p <* 0.01). Depressive symptoms were negatively correlated with cognitive function (*r*=-0.202, *p <* 0.01).

**Table 2:** Bivariate correlation matrix for healthy lifestyles, depressive symptoms, and cognitive function

Variable	Healthy Lifestyles	Depressive symptoms	Cognitive function
Healthy Lifestyles	1		
Depressive symptoms	-0.102^***^	1	
Cognitive function	0.084^***^	-0.202^***^	1

Note^***^p < 0.01

**Fig. 2:**
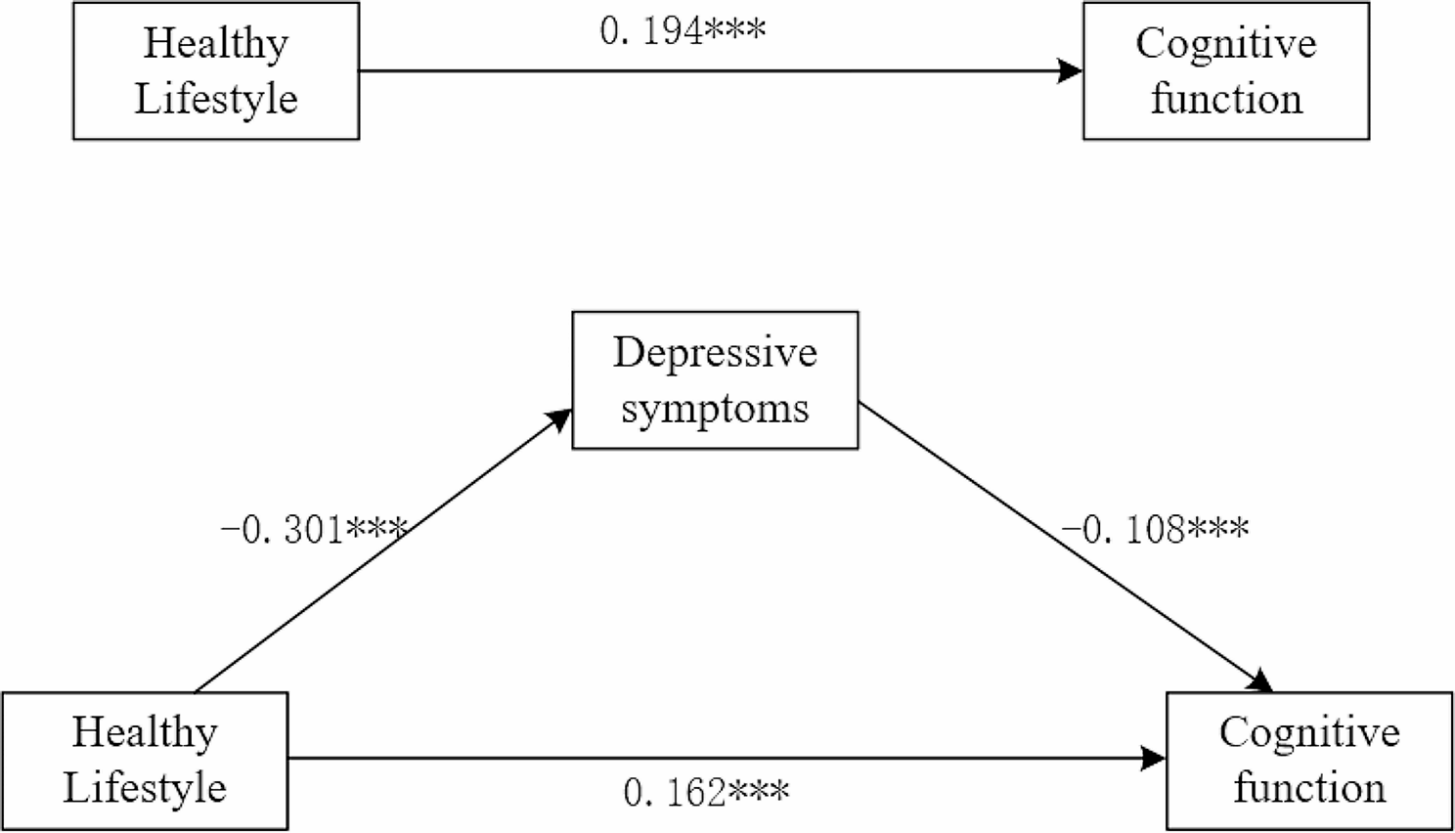
Relationship between healthy lifestyles, depressive symptoms, and cognitive function

Regression analysis was utilized to explore the mediating role of depressive symptoms on the relationship between healthy lifestyles and cognitive function. We calculate the variance inflation factor (VIF) of the covariates of the three equations separately, and the corresponding variance inflation factors (VIF) of all the variables are < 2, indicating that there is not excessive multicollinearity problem [48, 49]. Table 3; Fig. 2 demonstrate the results of the mediation analysis of depressive symptoms between healthy lifestyles and cognitive function after controlling for covariates. In the total effects regression, healthy lifestyles were a significant predictor of cognitive function (*β* = 0.194, *p <* 0.01). This relationship remained significant when depressive symptoms was considered (*β* = 0.162, *p <* 0.01). Moreover, healthy lifestyles showed a significant effect on depressive symptoms (*β*=-0.301, *p <* 0.01), whereas depressive symptoms exerted a significant effect on cognitive function (*β*=-0.136, *p <* 0.01).

**Table 3:** The mediating role of depressive symptoms between healthy lifestyles scores and cognitive function

Variable	Cognitive function	Depressive symptoms		Cognitive function
Healthy Lifestyles	0.194^***^		-0.301^***^	0.162^***^
	(0.113, 0.275)		(-0.400, -0.203)	(0.081, 0.243)
Age	-0.138^***^		0.020^***^	-0.136^***^
	(-0.148, -0.128)		(0.011, 0.029)	(-0.145, -0.126)
Gender	0.751^***^		-0.569^***^	0,689^***^
	(0.580, 0.921)		(-0.750, -0.387)	(0.519, 0.860)
Ethnic	-0.585^***^		-0.020	-0.587^***^
	(-0.886, -0.284)		(-0.373, 0.333)	(-0.886, -0.288)
Hukou	0.228^**^		-0.052	0.222^**^
	(0.030, 0.425)		(-0.262, 0.157)	(0.026, 0.418)
Marriage	0.538^***^		-0.345^***^	0.501^***^
	(0.341, 0.735)		(-0.559, -0.132)	(0.305, 0.697)
Education	0.117^***^		-0.012	0.116^***^
	(0.097, 0.138)		(-0.035, 0.012)	(0.096, 0.137)
Socioeconomic status	0.203^***^		-0.957^***^	0.100
	(0.072, 0.334)		(-1.105, -0.809)	(-0.033, 0.233)
Living arrangement	-0.555^***^		-0.610^***^	-0.621^***^
	(-0.795, -0.315)		(-0.861, -0.359)	(-0.860, -0.382)
Self-rated health	0.538^***^		-2.062^***^	0.316^***^
	(0.442, 0.634)		(-2.166, -1.958)	(0.214, 0.418)
Depressive symptoms				-0.108^***^
				(-0.130, -0.085)
Constant	34.945^***^		17.438^***^	36.821^***^
	(34.046, 35.843)		(16.426, 18.450)	(35.841, 37.800)
Observations	8272		8272	8272
R-squared	0.251		0.254	0.261

*Note* 95% confidence intervals in brackets; ^*^p < 0.1, ^**^p < 0.05, ^***^p < 0.01

The Sobel and bootstrap tests were conducted to check for indirect, direct, and total effects [50]. As shown in Table 4, Sobel’s test indicates that the indirect effect (Z = 5.098, *p <* 0.001), direct effect (Z = 3.322, *p <* 0.001), and total effect (Z = 3.971, *p <* 0.001) were significant. It suggested that healthy lifestyles affect depressive symptoms, which in turn affects cognitive function. The bootstrap method indicated that the direct effect of healthy lifestyles on cognitive function was 0.162 (95% CI: 0.082, 0.241), while the total effect was 0.194 (95% CI: 0.113, 0.272). The indirect effect of healthy lifestyles on cognitive function mediated by depressive symptoms was 0.032 (95% CI: 0.020, 0.045). All paths were significant, indicating the robustness of the mediation model results [51].

**Table 4:** Sobel and Bootstrap tests for mediation models

Paths	Observed Coefficient	Sobel test		Bootstrap test			
		Z Value	P	Bootstrap Standard Error	P	LLCI	ULCI
Indirect effect	0.032	5.098	0.000	0.006	0.000	0.020	0.045
Direct effect	0.162	3.322	0.000	0.041	0.000	0.082	0.241
Total effect	0.194	3.971	0.000	0.041	0.000	0.113	0.272

*Note* LLCI, lower level for confidence interval; ULCI, upper level for confidence interval

## Discussion

Using data from the 2018 Chinese Longitudinal Healthy Longevity Survey (CLHLS), the study found significant associations between healthy lifestyles and depressive symptoms and cognitive function among Chinese older adults. Depressive symptoms partially mediated the relationship between healthy lifestyles and cognitive functioning. The Sobel and Bootstrap methods supported the results of the underlying analyses, indicating the robustness of the results.

The present study found that healthy lifestyles have a positive predictive effect on cognitive function in the Chinese elderly population, which supports hypothesis 1. Several potential mechanisms underpin this relationship. For instance, smoking can elevate oxidative stress levels, affecting the cognitive degenerative process and increasing the risk of developing age-related cognitive impairment [52]. Excessive alcohol consumption may result in alcohol-related brain damage, including alcohol-related memory deficits and executive dysfunction [53]. Additionally, there exists a favorable correlation between physical exercise and cognitive improvement [54]. Physical activity boosts the levels of brain-derived neurotrophic factors, which play a pivotal role in enhancing cognitive performance [55]. Different types of food provide various nutrients, such as vitamins, minerals, and antioxidants, closely linked to brain health and cognitive function [56]. Simultaneously, the physiological functions related to nutrient intake, such as chewing, salivation, and absorption, tend to diminish with age [57]. Therefore, older adults are more susceptible to poor dietary habits, potentially leading to cognitive impairment [58]. Furthermore, this study provides evidence regarding the collective impact of multiple healthy lifestyle factors on cognition, comprehensively evaluating them through assessments of smoking, alcohol consumption, exercise status, and nutrient intake [59]. Regular physical activity increases blood flow to the brain and promotes the release of nerve growth factors, which contribute to neuronal growth and survival [60]. Positive social interactions reduce stress, loneliness and depression and provide cognitive stimulation to keep the brain active [61]. Good quality sleep helps the brain clear metabolic waste such as beta-amyloid, and the accumulation of these waste products is associated with the development of cognitive disorders such as Alzheimer’s disease [62].

Healthy lifestyles are associated with a reduced risk of depressive symptoms in older adults, confirming Hypothesis 2. The connection between healthy lifestyles and depressive symptoms can be elucidated through various biological and social mechanisms. Smoking, for instance, induces a short-term pharmacological effect that stimulates the brain and alleviates stress and anxiety [63]. However, it’s important to note that smoking can be addictive and potentially render individuals more sensitive, vulnerable, and anxious. On the other hand, excessive alcohol consumption has the potential to lead to alcohol dependence, as it can alter neurotransmitter production and brain function, heightening feelings of anxiety [64]. In contrast, physical activity plays an effective role in stimulating regional cerebral blood flow and promoting the body’s dopamine production, thereby maintaining a positive mood [65]. Additionally, the consumption of a diverse and healthy diet is closely associated with better nutritional status, which, in turn, can contribute to the reduction of depressive symptoms [66]. Moreover, the social context significantly influences the connection between lifestyles and depressive symptoms. Social support, for example, has the potential to promote a healthy lifestyle by alleviating stress and providing essential emotional support, effectively reducing an individual’s risk of depressive symptoms [67]. Conversely, high-intensity social stress, such as work-related pressure and family conflicts, can lead to unhealthy lifestyles, including irregular sleep patterns, disrupted dietary habits, and a lack of physical exercise, consequently increasing the risk of depressive symptoms [68].

Depressive symptoms are associated with cognitive impairment, supporting Hypothesis 3. Indeed, depressive symptoms are associated with abnormal neurotransmitter secretion and altered neuronal activity. Depressed individuals typically exhibit reduced levels of neurotransmitters like serotonin and norepinephrine in their brains, along with elevated levels of excitatory neurotransmitters like glutamate [69]. These abnormal neurotransmitter levels can lead to unstable neuronal activity, subsequently impacting cognitive function [70]. Depressive symptoms are often accompanied by a deterioration in social functioning, leading to damage to social networks and support systems, which may result in a dearth of emotional support and social isolation [71]. Research indicates that decreased social functioning and weakened social networks are associated with cognitive impairment [72]. Moreover, individuals with depressive symptoms often experience cognitive impairments, including poor concentration and memory lapses [73]. Specifically, they often experience fatigue, helplessness, and disorientation, and these emotional states could impact their ability to concentrate and remember information. Furthermore, depressive symptoms are associated with lower self-evaluation and self-esteem, potentially influencing the performance of cognitive function [74]. Therefore, the mechanisms by which depressive symptoms affect cognitive functioning are multifaceted, including biological, sociological, and psychological mechanisms.

The results of the Sobel and Bootstrap analyses suggest that depressive symptoms partially mediate the relationship between healthy lifestyles and cognitive function. Specifically, the direct effect of healthy lifestyles on cognitive function was 0.162, the total effect was 0.194, and the indirect effect mediated by depressive symptoms was 0.032. These findings suggest that healthy lifestyles have the potential to deter the onset of depressive symptoms, thereby reducing the risk of cognitive decline in the elderly population [75]. Therefore, it is imperative to prioritize the well-being of older adults by focusing on their healthy lifestyles and mental health [76]. To enhance cognitive function in this demographic, we recommend the promotion of healthy lifestyles and the cultivation of positive psychological states. This can be accomplished through the reinforcement of physical exercise, the advocacy of a well-rounded and nutritious diet, and the provision of professional mental health support and counseling services [77]. Implementing these measures can prevent cognitive impairment and improve the quality of life of older adults, thus contributing to the alleviation of the significant challenges presented by population aging [78].

The interpretation of the results of this study should be considered in the context of several limitations. Firstly, this study is a cross-sectional design, which cannot establish precise causal relationships between variables. Future studies could employ longitudinal randomized controlled trials to verify causal relationships between variables. Secondly, the data on healthy lifestyles and depression were assessed through self-report questionnaires, which may introduce reporting biases. Subsequently, although this study controlled for several covariates, potential confounding factors may still exist and influence our conclusions. Finally, we excluded samples containing missing values, which could introduce potential estimation bias. Once again, the limitation about the sample being restricted to those who were able to answer the questionnaire is significant, and this constraint may introduce selection bias.

## Conclusion

This study investigated the relationship between healthy lifestyles, depressive symptoms, and cognitive function in Chinese older adults. Our findings showed significant correlations between healthy lifestyles and depressive symptoms and cognitive function in Chinese older adults. Depressive symptoms were found to mediate the relationship between healthy lifestyles and cognitive function. Our study suggests that relevant governments and staff should consider the mechanism of action of promoting healthy lifestyles and mental health diversion when developing strategies to improve cognitive functioning in older adults. Initiatives such as lifestyle programs, psychological support and counseling services, regular check-ups, and healthcare interventions specifically targeting older adults can effectively prevent or delay cognitive decline, thus achieving the goal of healthy aging.

## Data Availability

The data for this study was the Chinese Longitudinal Healthy Longevity Survey (CLHLS) by the Center for Healthy Aging and Development, Peking University. https://doi.org/10.18170/DVN/WBO7LK.
